# Supplementary material for: A novel speech emotion recognition method based on feature construction and ensemble learning
Source: PLoS One. 2022 Aug 15;17(8):e0267132. doi: 10.1371/journal.pone.0267132 (PMC9377622; doi:10.1371/journal.pone.0267132)
Supplement: S1 File — (PDF) [file pone.0267132.s001.pdf]

In editing this article, Mr. Xu Liang gave great support in English grammar, and Mr. Guo Yi provided support for the revision of professional terms.
