# Supplementary material for: A novel speech emotion recognition method based on feature construction and ensemble learning
Source: PLoS One. 2022 Aug 15;17(8):e0267132. doi: 10.1371/journal.pone.0267132 (PMC9377622; doi:10.1371/journal.pone.0267132)
Supplement: Attachment — Submitted filename: Response to Reviewers.pdf [file pone.0267132.s002.pdf]

Response to Reviewers

This letter that responds to each point raised by the academic editor and the two reviewers. Firstly, I thank the editorial department very much for its recognition of this research work and detailed modification opinions. Secondly, I am very grateful to the two reviewers for their hard work and detailed review of the report. All of the points raised by editor and reviewers are very important for me. So, following the points, we carefully revise this paper and provide some responses as follows.

Response to the academic editor. By carefully reading the modification suggestions and schemes put forward by the academic editor, we have revised the structure and submission process of the paper, which can be reflected in the new submission.

Response to the reviewer 1.

1. **The literature study of this paper is poor. It is recommended to consider the recent literautre and also provide the justification that how the proposed work is better over the existing ones.**

   In this paper, we propose a method to improve the level of human-computer interaction. The main purpose of this method is to improve the performance of speech emotion recognition. The references in this paper are not up-to-date, but conform to the research theme of this paper. At the same time, the experimental comparison of this paper is carried out for the data set CASIA (Chinese emotion corpus).

   According to the revision point, in CSCD and SCI database we searched the recent research of speech emotion recognition based on CASIA data set. The latest research literature are mostly based on neural network, and the model complexity is high and the performance is not very good. For example, the accuracy of a novel heterogeneous parallel revolution Bi LSTM for speech emotion recognition in CASIA data set is only 79.67%, the accuracy rate of a novel user emotional interaction design model using long and short term memory networks and deep learning on CASIA data set is only 72.5%, and the accuracy rate of speech emotion recognition based on transfer learning from the facenet framework (a) on CASIA data set is only 90%, the accuracy of attention based revolution skip bidirectional long short term memory network for speech emotion recognition in CASIA data set has only reached 72.5%. Therefore, we still insist on the effectiveness of the original references.

2. **The feature section (Section 3) need more detailed discussion.**

   In the section 3 (feature construction), firstly we introduced how to extract speech features (feature extraction), and then we introduced how to select subset from feature set (feature selection).

   In feature extraction subsection, we have referred to many literatures on speech signal processing, after experimental comparison (not appearing in the paper), we select the acoustic features in the paper and calculate the statistical characteristics of these acoustic features. How to calculate these acoustic features and their statistical characteristics are not detail discussed in the paper, because these calculation processes can be found in references.

   In feature selection subsection, we mainly introduced the feature selection method of

L-SFS, and the detailed calculation steps of this step are described in detail in this paper.

3. **Provide the citations for the datasets used in this work.**

Following the revision point, we add "CASIA Chinese emotional corpus was recorded by the Institute of Automation, Chinese Academy of Sciences. It includes four professional speakers and six kinds of emotions: anger, fear, happiness, neutral, sadness and surprise, a total of 7200 different pronunciation. 300 of the corpus are the same text. That is to say the same text given different emotions to read, these corpus can be used to compare the analysis of different emotional state of the acoustic and rhythmic performance." to the paper.

4. **The time complexity for the proposed algorithms to be estimated and compared using the existing models.**

The main research of this paper is to improve the performance of speech emotion recognition. There is no quantitative analysis of the time complexity of the model, but from the perspective of qualitative analysis, the time complexity of the model in this paper is more complex than a single classifier and simpler than the classifier based on neural network.

5. **There are several machine learning based classification algorithm but the authors studied very few in this paper. Why authors considered only few. Provide the justifications or refer. Machine learning algorithms for wireless senor networks: a survey for comparisons on various classification algorithms.**

We know that there are many classification models based on machine learning algorithms , such as linear regression, Bayesian classifier, decision tree, random forest, k-nearest neighbor, support vector machine, etc., but this paper selects k-nearest neighbor, support vector machine, xgboost and lightgbm. The reason is that after reading a lot of references, these four classifiers perform well in speech emotion classification, and the other classifiers do not perform well.

6. **Compare the model using recent existing algorithms.**

As the answer to point 1, most of the latest research is based on neural network, and its performance on CASIA data set is not as good as the method proposed in this paper. So, we decided not to add the modification of this part in this paper.

7. **List the limitations on proposed work.**

1) The research content proposed in this paper is for CASIA data set, which is for Chinese speech emotion recognition. So, there is a limitation of cross language emotional expression.
2) In this paper, the traditional acoustic feature extraction process reflects the human diligence and wisdom, but there is still not a complete feature set. Besides, the ensemble learning method achieves good results, but it is not clear that increasing (reducing) the number of single weak learner and changing the type of learner will improve the recognition accuracy. So, it remains be further tested.

Response to the reviewer 2.

1. **How does L-SFS help to extract and select features? This is missing.**

In the feature selection subsection, we have introduced the steps of L-SFS feature selection method in detail. In addition, in subsection 5.4, we have verified this method in the form of

experiments.

2. **The dataset CASIA needs to be explored.**

Following the revision point, we add “CASIA Chinese emotional corpus was recorded by the Institute of Automation, Chinese Academy of Sciences. It includes four professional speakers and six kinds of emotions: anger, fear, happiness, neutral, sadness and surprise, a total of 7200 different pronunciation. 300 of the corpus are the same text. That is to say the same text given different emotions to read, these corpus can be used to compare the analysis of different emotional state of the acoustic and rhythmic performance.” to the paper.

3. **The rationale for choosing SVM, KNN, LightGBM and XGBoost for constructing proposed Sklex model.**

We know that there are many classification models based on machine learning algorithms , such as linear regression, Bayesian classifier, decision tree, random forest, k-nearest neighbor, support vector machine, etc., but this paper selects k-nearest neighbor, support vector machine, xgboost and lightgbm. The reason is that after reading a lot of references, these four classifiers perform well in speech emotion classification, and the other classifiers do not perform well.

4. **The research questions to be addressed by the proposed work and research motivation need to be strengthed.**

5. **Why ensembling of four models give best results?**

Actually, we are not sure that selecting four classifiers will achieve the best performance. At present, the classification effect is ideal. If more classifiers are added, it may cause model flooding and increase the time complexity of the model; At the same time, reducing the classifier will reduce the performance of the model.

In the paper of Conclusion and Prospect section, we mentioned that “the ensemble learning method achieves good results, but it is not clear that increasing (reducing) the number of single weak learner and changing the type of learner will improve the recognition accuracy. So it remains be further tested.”, so we are not sure that choosing four classifiers will achieve the best performance.

6. **What are the hyper-parameters used for the experiment?**

Thank you very much for raising this question. We have made changes in the article, and the results are as follow tables.

Table 9. Super parameters of SVM.

probability	C	gamma	kernel
True	10	0.05	rbf

Table 10. Super parameters of XGBoost.

verbosity	random state	learning rate
1	0	0.3
max depth	n estimators	objective
5	200	multi:softmax

Table 11. Super parameters of LightGBM.

num leaves	objective	boosting type	learning rate	max depth	n estimators
10	multiclass	gbdt	0.1	16	100

Table 12. Super parameters of KNN.

algorithm	leaf size	n neighbors	p	weights
auto	5	20	1	uniform
